# Effects of Dietary Conjugated Linoleic Acid and Biopolymer Encapsulation on Lipid Metabolism in Mice

**DOI:** 10.3390/ijms14046848

**Published:** 2013-03-26

**Authors:** Sun Jin Hur, Doo Hwan Kim, Se Chul Chun, Si Kyung Lee

**Affiliations:** Department of Bioresources and Food Science, Konkuk University, 120 Neungdong-ro, Gwangjin-gu, Seoul 143-701, Korea; E-Mails: sjhur@konkuk.ac.kr (S.J.H.); kimdh@konkuk.ac.kr (D.H.K.); scchun@konkuk.ac.kr (S.C.C.)

**Keywords:** conjugated linoleic acid, biopolymers encapsulation, energy expenditure, adipose tissue, lipid metabolism

## Abstract

Forty mice were randomly divided into four groups on the basis of the diet to be fed as follows: 5% (low) fat diet (T1: LF); 20% (high) fat diet (T2: HF); 20% fat containing 1% conjugated linoleic acid (CLA) (T3: HFC); and 20% fat containing 1% CLA with 0.5% biopolymers (T4: HFCB). The high-fat with CLA diet groups (HFC and HFCB) and the low-fat diet group (LF) tended to have lower body weights and total adipose tissue weights than those of the high-fat diet group (HF). Serum leptin and triglyceride were significantly lower in the high fat with CLA-fed groups (HFC and HFCB) and the low-fat diet group (LF) than those in the high-fat diet group (HF). It is noteworthy that the high-fat with CLA and biopolymers group (HFCB) showed the lowest serum triglyceride and cholesterol concentrations. In the high-fat-fed group (HF), voluntary travel distance as a measure of physical activity decreased after three weeks of feeding. However, the CLA-fed groups showed increased physical activity. The groups fed high-fat diets supplemented with CLA alone and with CLA and biopolymers had higher viscosity of small intestinal contents than that in the low- and high-fat dietary groups.

## 1. Introduction

Obesity and being overweight can occur when energy intake is higher than energy expenditure. It has long been proposed that increased energy expenditure due to physical activity may be the most important factor in body weight control. Pescatello and VanHeest [1] reported that increase in obesity is due to a relatively greater decrease in physical activity rather than due to an increase in energy intake. Westerterp-Plantenga [2] reported that fat intake was related to a relatively lower satiety and that the fat content of food appears to affect meal frequency. In general, fat has a higher caloric density than proteins and carbohydrates, and its contribution to the palatability of foods promotes ingestion of calories [3]. Hill *et al.*[4] reported that consumption of a high-fat diet increases the total energy intake and that excess dietary fat is stored with greater efficiency than excess dietary carbohydrate or protein. Horton *et al.*[5] also reported that a greater proportion of excess energy is stored during fat overfeeding (approximately 90%–95%) than that with an equivalent amount of carbohydrate overfeeding (approximately 75%–85%). Thus, an increase in excessive energy intake from fat can reduce physical activity, and this decline of physical activity causes obesity [1]. Thus, it can be stated that energy sources play an important role in weight gain and obesity. In the meantime, Park *et al.*[6] first reported the influence of conjugated linoleic acid (CLA) on body composition in a study in which mice fed a diet with 0.5% CLA had decreased body fat mass and increased lean body mass. Numerous studies using various models, including human models, have reported the effect of CLA on body composition or fat reduction. Our previous study found that CLA supplementation decreased overall body weight and total fat and that the effect of dietary CLA on adipose tissue reduction was greater in an obese mouse model than that in a wild-type mouse model [7]. Despite the large number of studies showing that CLA increases energy expenditure, as shown by increased oxygen consumption [8–10], how CLA influences body weight or body fat in terms of different caloric and fat contents in animal models is poorly understood. Numerous studies have demonstrated that the ability of various biopolymers acting as dietary fibers, such as chitosan, pectin, guar gum, xanthan gum or modified starch, can lower blood cholesterol concentrations and reduce lipid absorption. Dietary fibers could decrease serum cholesterol concentrations by reducing the amount of absorbed cholesterol. Furthermore, dietary fibers may interfere with the formation of lipid droplets, or they may interfere with the ability of lipase to access the lipids contained within the lipid droplets. In an early study, Lairon [11] reported that dietary fibers can alter the breakup and coalescence of lipid droplets in the stomach and small intestine, thereby altering the surface area of emulsified lipid exposed to digestive enzymes. In the same study, the authors reported that various dietary fiber sources can bind to bile acids, as well as to mixed micelle components; thus, they explained the particle disruption of the micellization process, which leads to reduced micellar solubilization of lipids. For instance, cationic chitosan can bind to the surfaces of anionic lipid droplets stabilized by bile salt or phospholipids and reduce lipase activity by preventing lipase from coming into contact with emulsified lipid substrates [12]. However, how biopolymer encapsulation influences lipid metabolism in a CLA dietary model remains to be elucidated. In addition, there is little information on how dietary CLA influences lipid metabolism in models fed the same amount of calories. Thus, the purpose of this study was to determine the effects of CLA and biopolymers on lipid metabolism in mice fed high- and low-fat diets.

## 2. Results

### 2.1. Food Intake and Body Weight

**Figure 1:**
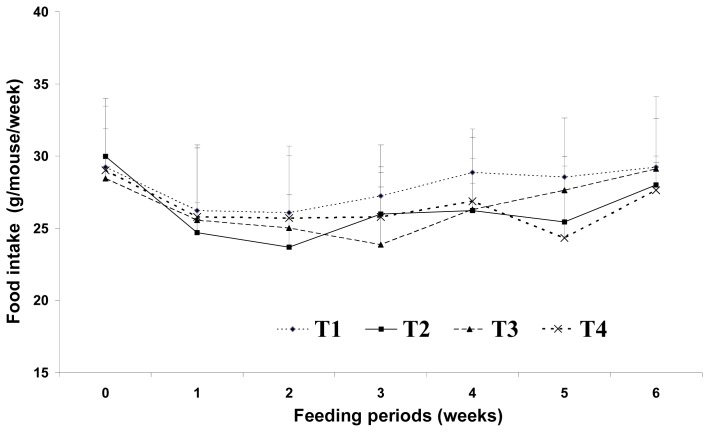
Effect of binding of conjugated linoleic acid with biopolymers on food intakes in mice fed high- and low-fat diets. Significance was set at *p* < 0.05.

**Figure 2:**
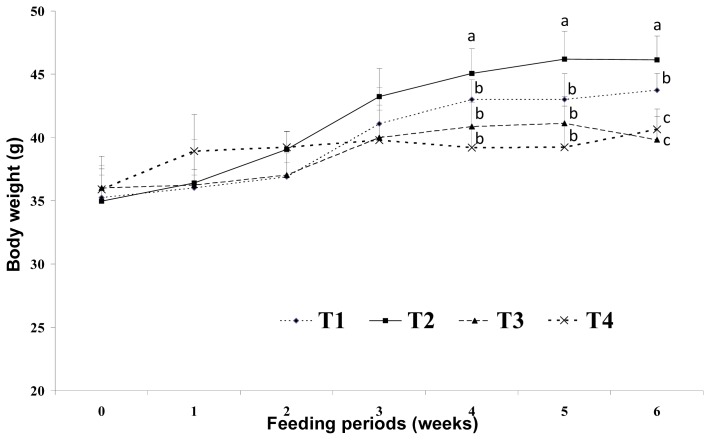
Effect of binding of conjugated linoleic acid with biopolymers on body weight in mice fed high- and low-fat diets. Significance was set at *p* < 0.05.

There were no significant differences in food intake among the dietary groups (Figures 1 and 2). After three weeks of feeding, the high-fat with CLA diet groups (HFC and HFCB) and the low-fat diet group (LF) tended to have lower body weights than that of the control group (HF). This result indicates that low- and high-fat diets with CLA can suppress body weight gain; this is despite the fact that total calorie levels were the same for both the low- (5%) and high-fat (5%) diet groups.

### 2.2. Adipose Tissue Weigh

**Figure 3:**
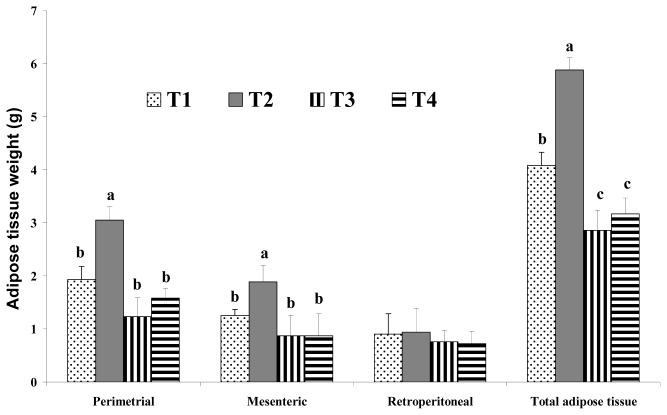
Effect of binding of conjugated linoleic acid with biopolymers on adipose tissue weights in mice fed high- and low-fat diets. Significance was set at *p* < 0.05.

Total adipose tissue weight was calculated as the sum of perimetrial, mesenteric and retroperitoneal adipose tissue weights (Figure 3). The group fed a high-fat CLA diet (HFC) and the group fed a high-fat CLA and biopolymer diet (HFCB) had significantly lower total adipose tissue weights than their control groups (LF and HF).

### 2.3. Blood Characteristics

Table 1 shows the effect of dietary CLA with biopolymers on blood characteristics. Serum leptin concentrations were significantly lower in the groups fed high-fat CLA diets (HFC and HFCB) and in the low-fat diet group (LF) than those in the high-fat diet group (HF); conversely, adiponectin was significantly higher in the CLA diet groups (HFC and HFCB) than that in the other dietary groups. Serum triglyceride and cholesterol concentrations were significantly lower in the high-fat diet with CLA groups (HFC and HFCB) than those in the control group (HF). It is noteworthy that the group fed the high fat with CLA and biopolymers diet (HFCB) had the lowest serum triglyceride and cholesterol concentrations among all the dietary groups. Glucose concentrations were significantly higher in the low-fat diet group (LF) than those in the high-fat diet groups (HF, HFC and HFCB).

### 2.4. Voluntary Travel Distance

**Figure 4:**
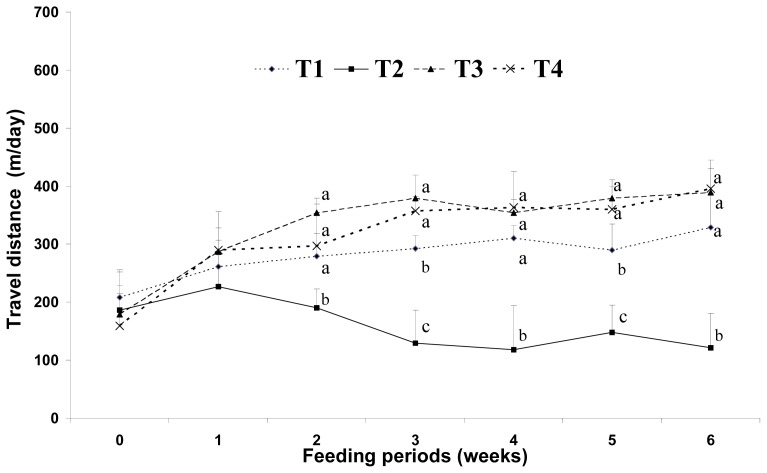
Effect of binding of conjugated linoleic acid with biopolymers on voluntary travel distance in mice fed high- and low-fat diets. Significance was set at *p* < 0.05.

Figure 4 shows the effect of a diet supplemented with CLA and biopolymers on voluntary travel distance. Voluntary travel distance as a measure of physical activity decreased after three weeks of feeding in the high-fat fed group (HF); however, the CLA-fed groups (HFC and HFCB) displayed a slight increase in physical activity, although the fat content of these groups was higher than that of HF. Increasing physical activity is an important strategy for preventing both weight gain and weight regain [13]. However, there is little scientific information as to how much physical activity is required to prevent weight gain in mice. In the present study, we found that a low-fat (5%) diet increases the voluntary travel distance compared to a high-fat (20%) diet. Mice fed a high-fat diet supplemented with CLA (HFC and HFCB) showed slightly higher levels of voluntary travel distance than mice in the other dietary groups.

### 2.5. Viscosity of Small Intestine Contents

**Figure 5:**
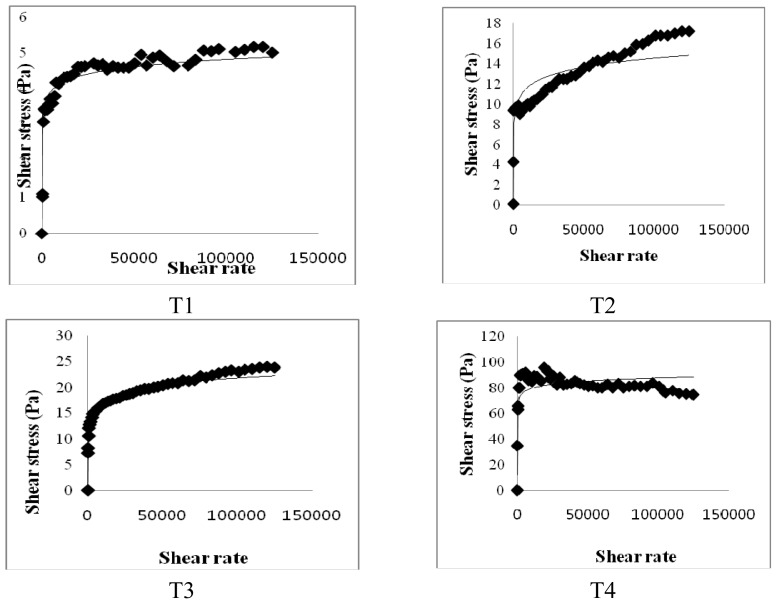
Effect of binding of conjugated linoleic acid with biopolymers on the viscosity of small intestine contents in mice fed high- and low-fat diets. Significance was set at *p* < 0.05.

Figure 5 shows the effect of dietary CLA with biopolymers on the viscosity of small intestine contents. The viscosity of small intestinal contents was higher in the 20% fat diet groups (HF, HFC and HFCB) than that in the 5% fat diet group (LF). The dietary CLA and biopolymers groups (HFC and HFCB) showed higher viscosity of small intestinal contents than that of the other dietary groups (LF and HF).

## 3. Discussion

Increased food intake and decreased energy expenditure may increase body weight. Westerterp-Plantenga [2] reported that the fat content of food appears to affect meal frequency. However, in the current study, we found no significant difference in food intake among the dietary groups. This may be because the total dietary calories were the same for the 5% and 20% fat diet groups. Sneddon *et al.*[14] reported that the average daily food intakes did not become significantly different until weeks three of experiment, indicating that palatability of the diets was not a major issue in CLA fed rats. Thus, changes in lipid metabolism resulting from dietary CLA and biopolymer encapsulation may not be the result of different food intake amounts in the present study. Moreover, CLA alone may have been responsible for the reduction of adipose tissue mass or lipid metabolism observed in this study. Westerterp-Plantenga [2] reported that energy intake from fat has a greater effect on body weight than energy from non-fat sources. Hill *et al.*[4] reported that consumption of a high-fat diet increases the total energy intake and that excess dietary fat is stored with greater efficiency than similar dietary carbohydrate or protein excess. In this study, increases in body weight may have been more influenced by fat sources than carbohydrate sources under the same calorie conditions. There are several possible mechanisms for this reduced body weight in response to dietary CLA. We assumed that the major body weight reducing effect in mice was the result of decreased adipose tissue weight. This may be closely related with increased energy expenditure caused by increased voluntary activity, such as travel distance. Park *et al.*[15] reported that dietary CLA can reduce adipose cell mass and/or cell numbers and can cause inhibition of stearoyl-CoA desaturase, as well as increased fatty acid β-oxidation in skeletal muscle. This increased fatty acid β-oxidation in skeletal muscle may be related to increased energy expenditure caused by dietary CLA. There are many possible mechanisms that may explain reduced body fat in response to CLA treatments. In an earlier study, Park *et al.*[6] reported that CLA increases fat oxidation and this is also supported by increased carnitine palmitoyltransferase activity in the fat pads, muscles and liver of CLA-fed mice. Those authors also reported triglyceride uptake and storage in adipose depots, as CLA has been shown to inhibit lipoprotein lipase activity in 3T3-L1 adipocytes [6]. Another possible mechanism for the reduced body fat seen after dietary CLA intake may be energy expenditure. Our findings in the present study agree with their study. In the present study, the levels of voluntary travel distance were significantly greater in CLA-fed mice than those in control group mice, regardless of the amount of fat. This increase in voluntary travel distance as a measure of physical activity caused by dietary CLA resulted in increased energy expenditure and adipose tissue depletion. This may be one of the main mechanisms involved in the fat reduction effect of CLA. However, we saw no significant difference in adipose tissue weight between the group fed with a high-fat CLA diet and the group fed with a high-fat CLA and biopolymer diet. This finding indicates that adipose tissue depletion may originate from CLA itself and not from biopolymers.

Leptin is a protein hormone with important effects on the regulation of body weight and lipid metabolism. It is secreted by adipocytes in proportion to the amount of lipid stored and may function as a signal of body energy stores to the brain [16]. Several studies have reported the effects of dietary CLA on leptin concentrations. Bhattachara *et al.*[17] suggested that CLA and exercise in combination may reduce serum leptin and peritoneal fat mRNA expression, which may explain, in part, the lowest change in fat mass in CLA/exercise mice. They also suggested that CLA may have a role in ameliorating the adverse effects of exercise on the immune response [17]. Sneddon *et al.*[14] reported that increased leptin transport into the brain should not result in the gene changes observed in the hypothalamus or in the increased food intake exhibited. In addition, circulating adiponectin, which acts centrally to increase food intake, is reduced by approximately 40% with CLA plus n-3 long chain polyunsaturated fatty acid supplementation [14]. In our previous study [7], we found that a diet supplemented with CLA reduced serum leptin concentrations in an obese murine model. The findings of these previous studies suggest that depletion in fat mass may be due to the association of CLA with reduced serum leptin concentrations. In this study, leptin concentrations were decreased by dietary CLA. This may result from the effect of CLA itself rather than its metabolites [15]. Thus, one of the main reasons for serum leptin depletion through dietary CLA supplementation in this study may be the lower adipose tissue content.

Brown *et al.*[18] reported that in human adipose tissue, the *trans*-10, *cis*-12 isomer of CLA decreases triacylglyceride concentrations. Baumgard *et al.*[19] reported that dietary CLA inhibits transcription of enzymes involved in *de novo* fatty acid synthesis, desaturation of fatty acids and triglyceride synthesis. Our findings in the present study agree with these results. In this study, serum glucose concentrations were not significantly different among the groups fed a high-fat diet (HF, HFC and HFCB). Conversely, Roche *et al.*[20] reported that *trans*-10, *cis*-12 supplementation in mice was associated with increased serum glucose and insulin concentrations, whereas the *cis*-9, *trans*-11 supplementation group showed no weight loss, but had lower concentrations of triglyceride and free fatty acids. Lee *et al.*[21] reported that the effect of CLA on blood glucose concentration was inconsistent. Thus, we assume that the effect of CLA on glucose may depend on various factors, such as mixture of CLA isomers, fat content in the diet, animal model, genotype and feeding period.

**Figure 6:**
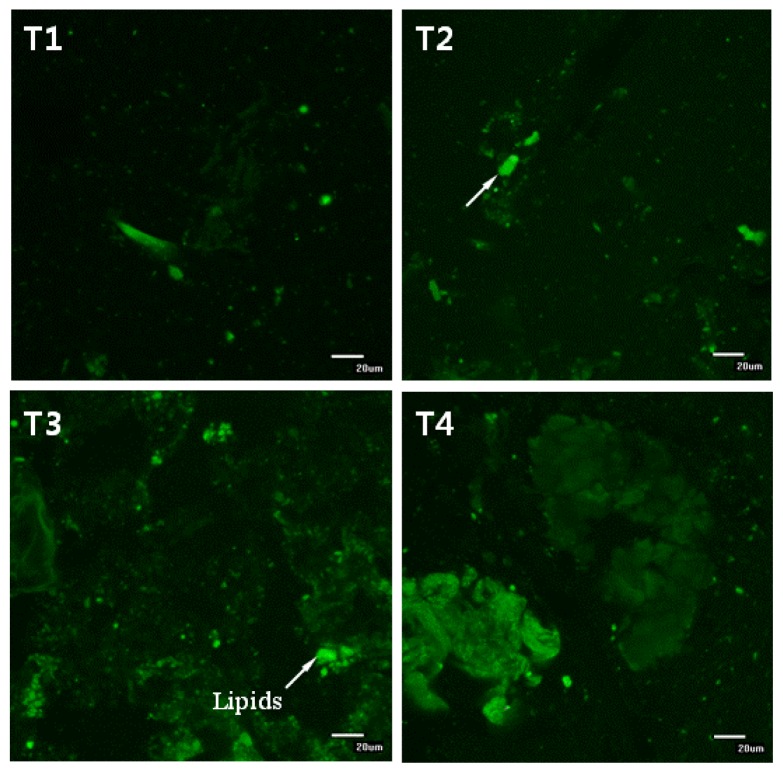
Confocal fluorescence images showing small intestinal contents after staining with Nile red. All green colored images represent stained lipids.

In this study, serum triglyceride and cholesterol concentrations decreased in mice fed a high-fat diet supplemented by CLA and biopolymers. In previous studies, Riserus *et al.*[22] found that HDL-cholesterol concentrations significantly decreased when the *tran*-10, *cis*-12 isomer was administered, but not when a mixture of the *trans*-10, *cis*-12 and the *cis*-9, *trans*-11 isomer was administered; in contrast, Terpstra [23] reported in a review that many studies did not show any significant effect of CLA on plasma cholesterol concentrations or on LDL-cholesterol concentrations. In this respect, we assume that cholesterol concentrations may be related to the isomers in the CLA mixture. In this study, serum triglyceride and cholesterol concentrations may have been influenced by biopolymers (pectin and chitosan) acting as dietary fibers. Dietary fibers may interfere with absorption of lipid droplets (with cholesterol) into the villi in the small intestine; alternatively, they may interfere with the ability of lipase to access the lipids contained within them. Beysseriat *et al*. [12] reported that the ability of dietary fibers to reduce cholesterol digestion and/or absorption through this mechanism depends on their ability to promote droplet aggregation or to adsorb lipid droplet on the surface, which strongly depends on the electrical charge, molecular weight and structure of fibers. In this study, lipid droplets in the diets were encapsulated with biopolymers (see Figure 6). Furthermore, the residual lipids in the small intestines of mice in the dietary CLA and biopolymer groups (HFC and HFCB) were higher than those in the control group (HF) (Figure 6). These results indicate that biopolymer encapsulation of lipids could decrease lipid digestion; thus, triglyceride and cholesterol concentrations in serum were decreased. Mun *et al.*[24] reported that cationic chitosan can form a polymer coating around lipid droplets that are stabilized by anionic surface-active materials, such as phospholipids and bile salt. In this study, biopolymer encapsulation around lipid droplets may have inhibited the ability of lipase or bile salt to access the surface of the lipid droplets, resulting in decreased digestion or absorption of triglycerides and cholesterol. Conversely, a high-fat diet supplemented with CLA (HFC) did not reduce blood cholesterol concentrations in this study. This result indicates that triglyceride and cholesterol reduction is mainly influenced by dietary supplementation with biopolymers and that dietary CLA is less influential. Thus, biopolymer supplementation can improve the effect of dietary CLA by inhibiting lipids, including triglycerides and cholesterol. However, our preliminary study (data not shown) revealed that small amounts of dietary CLA with biopolymers (less than 0.1%) did not decrease serum cholesterol and triglyceride concentrations, although dietary CLA did reduce the adipose tissue weight of mice. Therefore, we believe the amounts of both dietary CLA and biopolymers play an important role in lipid digestion and absorption.

Earlier studies have suggested that energy sources have a large influence on physical activity and weight gain. Wells *et al.*[25] reported that behavior was different after fat and carbohydrate ingestion; fat seems to induce more restful feelings than carbohydrates. Horton *et al*. [5] reported that a greater proportion of excess energy is stored during fat overfeeding (approximately 90%–95%) than an equivalent amount of carbohydrate overfeeding (approximately 75%–85%). In this study, voluntary travel distance as a measure of physical activity was greater in the low-fat (5%) diet group (LF) than that in the high-fat (20%) diet group (HF). This may be because the low-fat diet in this study contained more carbohydrate sources than the high-fat diet groups under the same calorie conditions. The high-fat diet with CLA and biopolymers groups (HFC and HFCB) showed an increase in voluntary travel distance. This finding indicates that dietary CLA can influence physical activity without the intervention of biopolymers. It has been proposed that CLA reduces adiposity by elevating energy expenditure via increased basal metabolic rate, thermogenesis or lipid oxidation in animals [26]. Mizunoya *et al.*[27] also suggested that oxygen consumption increased in mice fed a diet containing 1% CLA for eight weeks. In this study, the dietary CLA groups (HFC and HFCB) showed increased voluntary travel distance compared with that in the other dietary groups (LF and HF). This result may be one of the main causes of the adipose tissue depletion effect by dietary CLA. However, in this study, dietary supplementation with biopolymers did not have a significant influence in voluntary travel distance as a measure of physical activity. From the results of this study, we assume that dietary CLA and biopolymers do not exert negative effects on physical activity in a mice model.

In the present study, the viscosity of small intestinal contents may be associated with lipid digestion and absorption in the small intestine; in addition, differences in the viscosity of small intestinal contents would cause impairment in the hydrolysis and solubilization of lipids [28]. Meyer and Doty [29] also reported that high viscosity of the contents of the small intestine may delay lipid digestion, promoting absorption in the more distal part of the small intestine. An increase in solution viscosity is responsible for a decrease in the transit time of ingested food in the gastrointestinal tract. Therefore, an increase in solution viscosity causes a decrease in the transport rate of lipid digestion products present within micelles to the gastrointestinal walls. Thus, an increase in viscosity resulting from dietary CLA with biopolymers causes a reduction in the digestion and absorption rate of lipids. In general, the ability of soluble dietary fibers to increase solution viscosity, retard molecular diffusion and bind to water depends mainly on their molecular weight, conformation and interactions. Lairon [11] reported that dietary fibers can alter the breakup and coalescence of lipid droplets in the stomach and small intestine, thereby altering the surface area of lipids exposed to digestive enzymes. In Figure 6, undigested lipids in the small intestines of our mice models appear to be higher in dietary high fat in the CLA and biopolymers groups (HFC and HFCB) than that in the other groups (LF and HF). This finding indicates that dietary CLA with biopolymers reduced lipid digestion or absorption. A large amount of undigested lipid content in the small intestine due to the presence of dietary CLA and biopolymers may be the main reason for the increased viscosity of small intestine contents in those groups. In this study, adipose tissue weight was not significantly different between the high-fat diet with CLA group (HFC) and the high-fat diet with CLA and biopolymers group (HFCB). Conversely, the concentrations of triglyceride and cholesterol were lower in the high fat with CLA and biopolymers group (HFCB) than those in the high fat with CLA group (HFC). This result indicates that biopolymers are the main influence on serum triglyceride and cholesterol concentrations, whereas biopolymers may have less influence on adipose tissue weight or body weight.

## 4. Experimental Section

### 4.1. Materials

CLA mixture used in this study contained 63% *trans*-10, *cis*-12 CLA, 26% *cis*-9, *trans*-11 CLA and 7% other. CLA isomers (>96% pure) were purchased from Matreya (Pleasant Gap, PA, USA). Chitosan, pectin, Tween 20, acetic acid, sodium acetate, sodium chloride, monobasic sodium phosphate, dibasic sodium phosphate and Nile red were purchased from Sigma-Aldrich Chemical Company (St. Louis, MO, USA). Serum triglyceride, cholesterol and glucose assay kits were purchased from Thermo Electron, Inc. (Louisville, CO, USA).

### 4.2. Animal and Experimental Diet

Forty female ICR mice (age, 3 months; weight, 25–30 g) and a semi-purified powdered diet were purchased from Harlan Teklad (Madison, WI, USA). Isocaloric diet formulations were designed by Harlan Teklad (Madison, WI, USA). Animals were housed in individual, wire-bottomed cages in a windowless room with a 12-h light/dark cycle, under a protocol approved by the Animal Care Committee of Konkuk University. Food and water were provided *ad libitum* throughout the experiment. Fresh food was given on days 0, 2 and 5. After 2 weeks of adaptation to the environment and voluntary travel distance testing, 40 animals were randomly divided into 4 groups and fed the following diets: 5% fat (T1: LF); 20% fat (T2: HF); 20% fat containing 1% CLA (T3: HFC); and 20% fat containing 1% CLA with 0.5% biopolymer per kg (T4: HFCB). All groups were fed their respective diets for 6 weeks. The formulation of the experimental diets and treatment groups are listed in Table 2.

### 4.3. Biopolymer Encapsulation Preparation

Ten mass percentage of chitosan was dissolved in acetate buffer solutions (100 mM acetic acid: sodium acetate, pH 3.0, 0–150 mM NaCl). Ten mass percentage of pectin was dissolved in phosphate buffer solutions (2 M monobasic sodium phosphate and 2 M dibasic sodium phosphate, pH 7.0). These solutions were stirred for 12 h and then mixed for 3 h using a magnetic stirrer. During mixing, 1 mL of Tween 20 (0.1%, pH 7.6) was added dropwise to reduce surface tension and enhance formation of encapsulation. Biopolymer encapsulation was prepared by mixing a final volume of 10 wt% biopolymer solution and CLA together for 1 h using a bio-homogenizer. The mixture was continuously stirred for 15 min using power ultrasound at a frequency of 10 MHz, and this was then mixed with the experimental diets (final volume: 1% CLA mixed with 0.5% biopolymers in diet). This process was aimed at developing a coating layer around the lipophilic CLA. Encapsulation of biopolymers and CLA solution was confirmed using confocal microscopy (Figure 6).

### 4.4. Food Intake, Body Weight and Adipose Tissue Weight

Food intake and body weight gain were measured weekly, and adipose tissues were weighed after CO_2_ asphyxiation sacrifice.

### 4.5. Leptin, Adiponectin, Triglyceride, Cholesterol and Serum Glucose Concentrations

Blood was collected by cardiac vessel bleeding from anesthetized mice. Serum was obtained by centrifugation at 3,000 × *g* for 15 min at 4 °C. Serum leptin and adiponectin concentrations were measured using ELISA kit (R & D Systems, Inc., Minneapolis, MN, USA) techniques, as specified by the manufacturers. Serum triglyceride, cholesterol and glucose concentrations were measured using the enzymatic assay kits (Thermo Electron, Inc., Louisville, CO, USA), as per the manufacturers’ instructions.

### 4.6. Voluntary Travel Distance

Voluntary travel distance as a measure of physical activity was determined using wheel running systems. Mice were placed in an individual case (35 × 20 × 15 cm) containing a running wheel with a diameter of 10 cm under a 12-h light/dark cycle. After a period of familiarization (1 week), mice were allowed to run freely on the wheels. Data were collected daily for a period of 6 weeks. Voluntary travel distances were calculated through the use of the National Instruments Compact-DAQ, an NI 9411 module and a custom-designed LabVIEW program, which converted wheel revolution data to daily voluntary travel distance.

### 4.7. Viscosity of Small Intestinal Contents

Small intestinal contents were collected by gentle finger stripping. The small intestinal contents were pooled per tube. The viscosity of small intestinal contents was determined by the modified method of Turabi *et al.*[30]. The viscosity was measured using a rotational rheometer (Physica MCR 300, Stuttgart, Germany) in a system of coaxial cylinders (25 mm in diameter). A total of 200 μL of small intestinal contents was placed on a temperature control plate (37 °C) and shear stress and shear rate were then measured for 1 min.

### 4.8. Confocal Laser Scanning Microscopy

Small intestinal contents were collected by gently finger stripping. The small intestinal contents and biopolymer encapsulation were analyzed through confocal microscopy. A confocal scanning fluorescence microscope (Carl Zeiss, LSM 5 Live, GmbH, Jena, Germany) with a 20× objective lens was used to capture confocal images. Nile red (a lipid fluorescent dye) was excited with a 488-nm argon laser line. The fluorescence emitted from the sample was monitored using a fluorescence detector (543 nm) with a pinhole size of 150 μm. The resulting images consisted of 512 × 512 pixels, with a pixel size of 414 nm and a pixel dwell time of 5 s.

### 4.9. Statistics

The influences of dietary CLA and biopolymer encapsulation on lipid metabolism in mice fed high- and low-fat-fed diets were analyzed using SAS software (SAS Inst. Inc., Cary, NC, USA, 2001) by the generalized linear model procedure. The Student-Newman-Keuls multiple range test was used to compare differences between means.

## 5. Conclusions

From the results of this study, we conclude that the increase in the voluntary travel distance as a measure of physical activity resulting from dietary supplementation with CLA may be one of the main reasons for lipid depletion. Groups of mice fed a high-fat diet supplemented with CLA and biopolymers showed higher viscosity of small intestinal contents than the controls. This increase in viscosity may be one of the main reasons for triglyceride and cholesterol depletion. Thus, we assume that the lipid depletion effect of CLA may be accelerated by the addition of biopolymers. However, the nature of the relationship between increased physical activity and dietary CLA remains to be elucidated. The effect of CLA on lipid metabolism could be influenced to a large degree by the mixture of CLA isomers, CLA contents (or purity), dietary period or subjects. Therefore, future studies are needed to investigate how dietary CLA influences physical activity in animal models and how dietary supplementation with biopolymers is related to changes in lipid metabolism. The nature of the interaction between CLA and biopolymers (e.g., encapsulation efficiency and CLA encapsulation rate) also requires further investigation.

## Figures and Tables

**Table 1:** Effect of binding of conjugated linoleic acid with biopolymers on blood characteristics in mice fed high- and low-fat diets.

	Test items				
	
Treatments	Leptin (ng/mL)	Adiponectin (μg/dL)	Triglyceride (mg/dL)	Cholesterol (mg/dL)	Glucose (mg/dL)
T 1	72.12 b,c ± 4.33	11.69 b ± 4.80	112.24 c ± 11.29	150.56 b ± 11.87	57.24 a ± 2.31
T 2	118.36 a ± 3.37	16.41 b ± 6.09	179.17 a ± 10.23	176.64 a ± 12.36	50.50 b ± 1.23
T 3	62.23 c ± 6.42	30.36 a ± 4.23	151.46 b ± 9.45	168.38 a ± 10.19	48.36 b ± 4.97
T 4	77.75 b ± 7.37	25.22 a ± 5.37	120.27 c ± 13.78	122.71 c ± 9.97	51.78 b ± 3.45

a–d Means with different superscripts in the same column are significantly different (*p* < 0.05).

**Table 2:** Experimental diets and treatment group.

Items	Treatments 1			

	T1 5% fat	T2 20% fat	T3 20% fat with 1% CLA	T4 20% fat with 1% CLA +0.1% biopolymers
Casein	202	249.50	247.01	247.01
l-cystine	3.03	3.69	3.65	3.65
Corn starch	409.96	158.82	157.21	157.21
Maltodextrin	133	151.50	149.99	149.99
Sucrose	101	123.20	121.97	121.97
Soybean oil	50.50	202	199.98	199.98
Cellulose	50.50	50.50	50	45
Mineral Mix	35.35	43	42.57	42.57
Vitamin Mix	10.10	12.32	12.20	12.20
Calcium Phosphate Dibasic	2.02	2.42	2.40	2.40
Choline Bitartrate	2.53	3.03	3.00	3.00
TBHQ (antioxidant)	0.01	0.02	0.02	0.02
CLA	0	0	10	10
Biopolymers	0	0	0	5
Total	1,000	1,000	1,000	1,000

1 Different fat contents and the same total calorie among the diets. Isocaloric diets designed by Harlan Teklad (Madison, WI, USA).

CLA, conjugated linoleic acid; TBHQ, tert-Butylhydroquinone.
